# Supplementary material for: A liver immune rheostat regulates CD8 T cell immunity in chronic HBV infection
Source: Nature. 2024 Jul 10;631(8022):867–75. doi: 10.1038/s41586-024-07630-7 (PMC11269190; doi:10.1038/s41586-024-07630-7)
Supplement: Supplementary file 2 — Reporting Summary [file 41586_2024_7630_MOESM2_ESM.pdf]

Corresponding author(s): Percy A. Knolle
Last updated by author(s): May 5, 2024

# Reporting Summary

Nature Portfolio wishes to improve the reproducibility of the work that we publish. This form provides structure for consistency and transparency in reporting. For further information on Nature Portfolio policies, see our Editorial Policies and the Editorial Policy Checklist.

## Statistics

For all statistical analyses, confirm that the following items are present in the figure legend, table legend, main text, or Methods section.

| n/a | Confirmed | |
|---|---|---|
| ☐ | ☒ | The exact sample size (*n*) for each experimental group/condition, given as a discrete number and unit of measurement |
| ☐ | ☒ | A statement on whether measurements were taken from distinct samples or whether the same sample was measured repeatedly |
| ☐ | ☒ | The statistical test(s) used AND whether they are one- or two-sided<br>*Only common tests should be described solely by name; describe more complex techniques in the Methods section.* |
| ☐ | ☒ | A description of all covariates tested |
| ☐ | ☒ | A description of any assumptions or corrections, such as tests of normality and adjustment for multiple comparisons |
| ☐ | ☒ | A full description of the statistical parameters including central tendency (e.g. means) or other basic estimates (e.g. regression coefficient) AND variation (e.g. standard deviation) or associated estimates of uncertainty (e.g. confidence intervals) |
| ☐ | ☒ | For null hypothesis testing, the test statistic (e.g. *F*, *t*, *r*) with confidence intervals, effect sizes, degrees of freedom and *P* value noted<br>*Give P values as exact values whenever suitable.* |
| ☒ | ☐ | For Bayesian analysis, information on the choice of priors and Markov chain Monte Carlo settings |
| ☐ | ☒ | For hierarchical and complex designs, identification of the appropriate level for tests and full reporting of outcomes |
| ☒ | ☐ | Estimates of effect sizes (e.g. Cohen's *d*, Pearson's *r*), indicating how they were calculated |

*Our web collection on statistics for biologists contains articles on many of the points above.*

## Software and code

Policy information about availability of computer code

Data collection

The following machines were used for data collection for the respective application:

Flow cytometry: SP6800 (Sony Biotechnology); CytoFLEX S (Beckman Coulter)

Fluorescence activated cell sorting: SH800 (Sony Biotechnology); MoFlo Astrios EQ (Beckman Coulter); FACSMelody Cell Sorter (BD Biosciences); BD SORP FACS Aria (BD Biosciences)

Real-time impedance based cytotoxicity measurement: xCelligence RTCA MP device (ACEA Biosciences)

Confocal microscopy: TCS SP8 (Leica)

qRT-PCR: LightCycler 480 (Roche)

in vivo imaging: IVIS Lumina LT-Series III (Perkin Elmer)

sALT measurement: Reflotron®plus system (Roche)

HBeAg measurement in peripheral blood: ArchitectTM platform and the HBeAg reagent kit (Ref.: 6C32-27) with HBeAg quantitative calibrators (Ref.: 7P24-01, all: Abbott Laboratories)

Immunohistochemistry: Bond MAX, Bond Rxm, Aperio (Leica Biosystems)

Next Generation Sequencing: NextSeq 500 (Illumina); HiSeq2500 (Illumina); HiSeq3000 (Illumina); NovaSeq6000 (Illumina); MiSeq system (GenDX)

Data analysis

The following software, tools, packages and algorithms were used for data analysis:

Aperio Image Scope v12.4.0 (Leica); QuPath (v0.2.3); FlowJo v10.7.1 & v10.8.0 (BD); Imaris v9.6 (Bitplane); Drop-seq v1.12 pipeline (https://github.com/broadinstitute/Drop-seq); DESeq2 R package v2.1.28.1; GREIN DB v1; ggplot2 R package v3.3.2; prcomp function R v3.6.1; ggplot 2 & ggrepel R package v0.9.4; R v4.1.2 with Seurat package v4.3.0; Limma R package v3.58.1; GSEA v4.0.3; Python v3.12 custom script (https://zenodo.org/records/11040043); Cytoscape v3.7.1; igraph R package v2.0.2 (https://igraph.org/); bcl2fastq software v2.20.0.422; snakemake pipelines (https://gitlab.lrz.de/ImmunoPhysio/bulkSeqPipe);
Trimmomatic v0.36; STAR v2.5.3a; htseq v0.9.1; DESeq2 v1.24.03a; scPipe R package workflow v1.12.0, Seurat R package v3.2.0; pySCENIC pipeline v0.10.10; Harmony v1.2.0; clustree R package v0.4.0; UCELL R package v1.2.4

For further details please see methods section.

For manuscripts utilizing custom algorithms or software that are central to the research but not yet described in published literature, software must be made available to editors and reviewers. We strongly encourage code deposition in a community repository (e.g. GitHub). See the Nature Portfolio guidelines for submitting code & software for further information.

## Data

Policy information about availability of data

All manuscripts must include a data availability statement. This statement should provide the following information, where applicable:

- Accession codes, unique identifiers, or web links for publicly available datasets
- A description of any restrictions on data availability
- For clinical datasets or third party data, please ensure that the statement adheres to our policy

Data from mouse RNAseq are deposited at GEO accession number:
GSE168096 (https://www.ncbi.nlm.nih.gov/geo/query/acc.cgi?acc=GSE168096 token: yhwvqmmaxhudjqh)
GSE212925 (https://www.ncbi.nlm.nih.gov/geo/query/acc.cgi?acc=GSE212925 token: qxylgomkrhivfwx)
GSE214151 (https://www.ncbi.nlm.nih.gov/geo/query/acc.cgi?acc=GSE214151 token:wzyteyoyhzmntqx)

PRJEB36998 (https://www.ebi.ac.uk/ena/browser/view/PRJEB36998, published in Sandu et al., 2020, Cell reports)

Single-cell RNA-sequencing data of CD8 T cells from liver explants: https://figshare.com/s/9db8a1f1de89c1e4f18c
Single cell RNA-sequencing data of HBc-specific CD8 T cells from patients with chronic Hepatitis B virus infection: https://figshare.com/s/245d38cb7c4901b70b3f

Publicly available data sets:

GSE47045 (ref17); GSE70813 (ref19); MsigDB BIOCARTA dataset (https://www.gsea-msigdb.org/gsea/msigdb/); Molecular Genome Informatics database (http://www.informatics.jax.org/go/term/GO:0019722); http://amp.pharm.mssm.edu/Harmonizome (ref21); human gene database GeneCards (https://www.genecards.org/; TF checkpoint database (ref23); Eukaryotic promoter database & UCSC (GRCm38/mm10) database (ref24, https://genome.ucsc.edu/cgi-bin/hgTrackUi?db=mm10&c=chrX&g=encode3RenEnhancerEpdNewPromoter); JASPAR core database (ref25); HOCOMOCO database (ref26); GRCm38 reference genome ENSEMBL (annotation release #75, #91); human genome (version GRCh38); human CD8 T cell signatures from ref33

For further details please see methods section.

## Human research participants

Policy information about studies involving human research participants and Sex and Gender in Research.

Reporting on sex and gender

Human patient data (persistent HBV infection): PMBCs were collected from female patients (sex). Data on gender was not collected.

Population characteristics

Human patient data (persistent HBV infection): PBMCs were collected from patients with a age range from 29 to 53 years who were diagnosed for chronic Hepatitis B virus infection, naive to therapy, HBeAg negative, anti-HBs antibody negative, anti-HBe antibody negative, ALT [U/ml] in a range from 19-46, viral load [IU/mL] ranging from 31-1795.
Human research participants with hepatitis B virus infections whose liver CD8 T cells were analysed after fine-needle aspriation were stratified according to their hepatitis and HBeAg status as outlined in Fig.3e.

Recruitment

Patients with chronic hepatitis B for PBMC collection were recruited after giving informed consent to participate in this study. Patients with chronic hepatitis B or patients who had cleared the infection were recruited from outpatient clinics at the different sites for analysis of circulating or hepatic HBV-specific CD8 T cells and were included in the study based on the disease state (ongoing viral hepatitis with detection of viral markers and high ALT levels, anti-HBe+ infection with detection of viral markers and low ALT levels and clearance of infection with loss of viral markers and absence of increased ALT levels).

| | |
|---|---|
| Ethics oversight | Immunohistochemistry of human liver tissue was conducted according to federal guidelines, local ethics committee regulations of the Technical University of Munich, Germany (No 518/19 S-SR). Isolation of PBMCs from patients was conducted according to federal guidelines, local ethics committee regulations of Albert-Ludwigs-Universität, Freiburg, Germany (no. 474/14). Liver fine-needle-aspirations (FNAs) were collected from participants living with CHB at the Erasmus MC University Medical Center (Rotterdam, The Netherlands), the Toronto General Hospital (Toronto, Canada), and the Massachusetts General Hospital (Boston, USA). All participants provided written informed consent. This study was approved by institutional review boards at all 3 sites and was conducted in accordance with both the declaration of Helsinki and Istanbul. |

Note that full information on the approval of the study protocol must also be provided in the manuscript.

# Field-specific reporting

Please select the one below that is the best fit for your research. If you are not sure, read the appropriate sections before making your selection.

☒ Life sciences ☐ Behavioural & social sciences ☐ Ecological, evolutionary & environmental sciences

For a reference copy of the document with all sections, see nature.com/documents/nr-reporting-summary-flat.pdf

# Life sciences study design

All studies must disclose on these points even when the disclosure is negative.

| | |
|---|---|
| Sample size | Pilot experiments were used to estimate the sample size such that an appropriate statistical test could yield significant results. The exact n numbers used in this study are indicated in the each figure legend. |
| Data exclusions | Every mouse infected with an adenovirus or uninfected control mice was included in the data analysis. The acquired data of an individual (human or mouse) was only excluded if technical problems during sample processing or data acquisition occurred. |
| Replication | All experiments were repeated several times, details are given in each figure legend. |
| Randomization | Murine experiments: all mice were randomly assigned into experimental groups and treated accordingly.<br>The allocation of patients with chronic hepatitis B occurred based on the presence of viral markers and immune response markers (anti-HBe) and ALT levels. |
| Blinding | Bioinformatic analyses, histological analyses and analyses of confocal images were performed with code-labeled samples. Human samples were pseudonomised for all further processing. Researchers were not blinded for treatment or genotypes of mice to avoid mix-up of samples and handling by several scientists and research associates, experimental design and appropriate controls ensured accuracy and reproducibility of measurements and analyses. |

# Reporting for specific materials, systems and methods

We require information from authors about some types of materials, experimental systems and methods used in many studies. Here, indicate whether each material, system or method listed is relevant to your study. If you are not sure if a list item applies to your research, read the appropriate section before selecting a response.

## Materials & experimental systems

| n/a | Involved in the study | |
|---|---|---|
| ☐ | ☒ | Antibodies |
| ☐ | ☒ | Eukaryotic cell lines |
| ☒ | ☐ | Palaeontology and archaeology |
| ☐ | ☒ | Animals and other organisms |
| ☒ | ☐ | Clinical data |
| ☒ | ☐ | Dual use research of concern |

## Methods

| n/a | Involved in the study | |
|---|---|---|
| ☒ | ☐ | ChIP-seq |
| ☐ | ☒ | Flow cytometry |
| ☒ | ☐ | MRI-based neuroimaging |

## Antibodies

| | |
|---|---|
| Antibodies used | The following antibodies (clone, dilution, supplier, catalogue number) were used for staining of mouse cells:<br>anti-CD8 (53-6.7, 1:250, Biolegend, #100752), anti-CD45.1 (A20, 1:200, Biolegend, #110722, #110704, #110748), anti-CXCR6 (SA051D1, 1:200, Biolegend, #151117, #151104, #151108, #151109, #151115), anti-CX3CR1 (SA011F11, 1:200, Biolegend, #149016, #149004, #149006), anti-CD44 (IM7, 1:200, Biolegend, #103036), anti-CD69 (H1.2F3, 1:100, Biolegend, #104530 or Thermo Fisher Scientific #63-0691-82), anti-TIM-3 (B8.2C12, 1:200, Biolegend, #134008 and ThermoFisher Scientific #12-2231-82), anti-TIGIT (1G9, 1:200, Biolegend, #142111), anti-IFN-g (XMG1.2, 1:200, Biolegend, #505808), anti-CD19 (1D3, 1:200, Biolegend, #152404), anti-CD335 (29A1.4, 1:200, Biolegend, #137606), anti-Lck pY394 (A18002D, 1:100, Biolegend, #933104), CD39 (Duha59, 1:200, Biolegend, |

#143805), anti-CD45.2 (104, 1:200, Biolegend, #109805), anti-CD3 (17A2, 1:200, Biolegend, #100217), anti-NK1.1 (PK136, 1:100, Biolegend, #108747), anti-CD4 (GK1.5, 1:200, Biolegend, 100449), anti-CD49a (HMa1, 1:200, Biolegend, #142606),anti-PD-1 (J43, 1:200, Thermo Fisher Scientific, #46-9985-82), anti-LAG-3 (eBioC9B7W, 1:200, Thermo Fisher Scientific, #12-2231-82, #406-2239-42), anti-Tox (TXRX10, 1:100, Thermo Fisher Scientific, #12-6502-82), anti-Granzyme B (GB11, 1:200, Thermo Fisher Scientific, #GRB04 and #GRB05), anti-TNF (MP6-XT22, 1:200, Thermo Fisher Scientific, #25-7321-82), anti-4-1BB (17B5, 1:100, Thermo Fisher Scientific, #48-1371-82), anti-CD25 (PC61.5, 1:200, Thermo Fisher Scientific, #48-0251-82), anti-Akt pS473 (SDRNR, 1:100, Thermo Fisher Scientific, #25-9715-42), anti-rabbit IgG Fab2 (1:500, Cell signalling, #79408), anti-pPKA (47/PKA, 1:5, BD Biosciences, #560205), anti-CD103 (goat polyclonal, 1:200, R&D Systems, #AF1990), anti-MHCII (M5/144.15.2, 1:200, Biolegend, #107636), anti-CD146 (ME9F1, 1:100, Miltenyi, #130-102-846) anti-CD335 (29A1.4, 1:200, Biolegend, #137606), anti-CD73 (TY/11.8, 1:200, Thermo Fisher Scientific, #48-0731-82).

For staining of human cells, the following antibodies (clone, dilution, catalog number, Lot number) were used:
anti-CD14 (61D3, 1:100, #A15453, Lot 2406638), anti-CD19 (HIB19, 1:100, #17-0199-42, Lot 2472560) (all Thermo Fisher Scientific), anti-CD45RA (HI100, 1:200, #304178, Lot #2327528), anti-CCR7 (1:20, G043H7, #353244, Lot B347205) (all Biolegend), anti-CD8 (RPA-T8, 1:200, #563795, Lot 9346411), and anti-GZMB (GB11, 1:100, #563388, Lot 3317967) (all BD Bioscience)

The following antibodies were used for immunohistochemistry:
1.5 µg/mL polyclonal anti-HBcAg (Origene, #AP08118PU-S); 0.4 ng/µl polyclonal anti-GFP (Fitzgerald, #70R-10652)

The following antibodies (clone, dilution, supplier, catalog number) were used for staining of tissue sections analysed by confocal immunofluorescence imaging:
anti-CD3 (clone 17A2, 1:200, Biolegend, #100240,), anti-CD45.1 (clone A20, 1:200, Biolegend, #110732), anti-CD146 (clone ME-9F1, 1:100, Miltenyi, #130-102-846), anti-I-A/I-E (MHC class II) (clone M5/114.15.2, 1:200, Biolegend, #107622) and anti-CD103 (goat polyclonal, 1:200, R&D Systems, #AF1990) followed by anti-goat IgG (donkey polyclonal, 1:500, Jackson ImmunoResearch, #705-625-147).

Validation

All antibodies listed in the previous section were validated by the manufacturer and/or by previous studies.

Information on the validation of antibodies for flow cytometry can be found as stated below:

Biolegend antibodies: https://www.biolegend.com/en-us/quality/quality-control
Biolegend employs a comprehensive approach to antibody validation, analyzing 1-3 target cell types with single- and multi-colour analysis to encompass positive and negative cell types. Upon confirming specificity, each new lot is required to match the intensity of the in-date reference lot, with the brightness (MFI) evaluated across both positive and negative populations to ensure consistency. Furthermore, quality control testing, including a series of titration dilutions, is conducted for every lot.

Thermo Fisher Scientific antibodies: https://www.thermofisher.com/de/de/home/life-science/antibodies/invitrogen-antibodyvalidation.html
Thermo Fisher Scientific tests each antibody using different methods, including flow cytometry, Immunoprecipitation-Mass Spectrometry Antibody Validation, Knockout and Knockdown Antibody Validation, Independent Antibody Validation, Peptide Array Antibody Validation, Cell Treatment, Neutralization Antibody Validation, Relative Expression Antibody Validation, and SNAP-ChIP Antibody Validation. The precise validation method for each antibody is outlined in its respective antibody datasheet.

BD Biosciences antibodies: https://www.bdbiosciences.com/en-eu/products/reagents/flow-cytometry-reagents/research-reagents/quality-and-reproducibility
BD Biosciences tests each antibody on primary cells, cell lines or transfectant models using different methods, including flow cytometry, immunofluorescence, immunohistochemistry, or western blot. The precise validation method for each antibody is outlined in its respective antibody datasheet.

Cell signalling antibodies: https://www.cellsignal.com/about-us/our-approach-process/antibody-validation-flow-cytometry
Flow-validated products undergo rigorous testing in biologically relevant models, ensuring specificity and an optimal signal-to-noise ratio (S/N) for both conjugated and unconjugated antibodies. Cross-platform validation further confirms antibody specificity. In addition, all antibodies have been tested for optimal dilution, specificity, stability and lot-to-lot reproducibility.

Miltenyi antibodies: https://www.miltenyibiotec.com/DE-en/products/macs-antibodies/antibody-validation.html
All antibodies are rigorously tested and validated before release. The precise validation method for each antibody is outlined in its respective antibody datasheet.

R&D Systems antibodies:https://www.rndsystems.com/products/rd-systems-approach-antibody-quality
Each antibody is manufactured under controlled conditions, undergoing rigorous quality control testing to ensure lot-to-lot consistency. Validation includes externsive specificity testing and testing of cross-reactivity using a variety of applications. he precise validation method for each antibody is
outlined in its respective antibody datasheet.

The anti-GFP and the anti-HBcAg antibodies were validated by the Institute of Pathology, School of Medicine, TUM.

# Eukaryotic cell lines

Policy information about cell lines and Sex and Gender in Research

Cell line source(s)

HEK293 cells (CRL-1573™) were obtained by ATCC, USA

Authentication | cell line was not authenticated

Mycoplasma contamination | Cell line was regularly tested for mycoplasma contamination and results were always negative.

Commonly misidentified lines (See ICLAC register) | *Name any commonly misidentified cell lines used in the study and provide a rationale for their use.*

## Animals and other research organisms

Policy information about studies involving animals; ARRIVE guidelines recommended for reporting animal research, and Sex and Gender in Research

**Laboratory animals**
6-8 week old C57Bl/6J male mice were purchased from Janvier or Charles River. H-2Kb(SIINFEKL)-restricted TCR-transgenic CD45.1+ mice, H-2Kb(MGLKFRQL)-restricted TCR-transgenic CD45.1+ mice (Ref: Isogawa 2013, PLOS Pathogens, doi:10.1371 journal.ppat.1003490, purchased from Charles River), and CD4-CrexICERfl/fl mice (B6.Cg-Tg(Cd4-cre)1Cwi x ICER-fl/fl) were bred under specific pathogen free conditions at TranslaTUM, Klinikum rechts der Isar. Mice were housed with a 12 h light - 12 h dark cycle. Temperature was set to 22+/-2 °C, humidity to 55+/-10% and checked daily.
Wild type littermates were used as controls as indicated.

**Wild animals**
No wild animals were used in this study.

**Reporting on sex**
In vivo experiments in mice were performed in male mice to have comparable virus to bodyweight ratios and quantitatively comparable antiviral immune responses.

**Field-collected samples**
No field-collected samples were used in this study.

**Ethics oversight**
Guidelines of the Federation of Laboratory Animal Science Association were implemented for breeding and experiments. Experiments were approved by the District Government of Upper Bavaria, Germany (permission numbers ROB-55.2-2532.Vet_02-14-185; ROB-55.2-2532.Vet_02-16-55, ROB-55.2-2532.Vet_02-18-100).

Note that full information on the approval of the study protocol must also be provided in the manuscript.

## Flow Cytometry

### Plots

Confirm that:

☒ The axis labels state the marker and fluorochrome used (e.g. CD4-FITC).

☒ The axis scales are clearly visible. Include numbers along axes only for bottom left plot of group (a 'group' is an analysis of identical markers).

☒ All plots are contour plots with outliers or pseudocolor plots.

☒ A numerical value for number of cells or percentage (with statistics) is provided.

### Methodology

**Sample preparation**
Isolation and culture of primary mouse cells:
Splenocyte isolation
Spleens were passed through a 100 µm cell strainer and red blood cells were lysed with Ammonium-Chloride-Potassium lysing buffer for 2 min.

Isolation of liver-associated lymphocytes
Before excision, livers were perfused with PBS via the portal vein. Livers were passed through 100 µm cell strainers and digested with 125 µg/mL collagenase type II (Worthington) in Gey´s balanced salt solution (GBSS, PAN Biotech) for 10 min at 37° C. For enrichment of liver-associated lymphocytes, a density gradient centrifugation with 40%/80% Percoll (GE Healthcare) was performed at 1440 x g for 20 min.

Isolation of primary mouse hepatocytes
Livers were perfused with 0.12 U/mL collagenase (SERVA) at 6 mL/min for 8 min via the portal vein. Livers were then removed, mechanically disrupted and passed through a 300 µm cell strainer. Liver cell suspensions were filtered through a 100 µm mesh and pelletised at 50 x g for 2 min. Hepatocytes were purified by density gradient centrifugation with 50%/80% Percoll (GE Healthcare) at 600 x g for 20 min. For cytotoxicity assays, 10,000 hepatocytes per well were seeded on 96 well E-plates (ACEA Biosciences) coated with 0.02% collagenR (SERVA). Cell attachment was achieved in supplemented William's E medium (PAN Biotech, 200 mM Glutamine (Thermo Fisher Scientific), 1 M Hepes pH 7.4, 104 U/mL Penicillin/Streptomycin, 50 mg/mL gentamycin (Merck), 0.005 ng/mL insulin (INSUMAN rapid, Sanofi), 1.6% DMSO (Merck) and 10% FBS (PAN Biotech). Attached cells were cultivated in supplemented William's E medium (as above) containing 1% FBS.

Isolation of primary mouse liver sinusoidal endothelial cells
Nonparenchymal liver cells were isolated from mouse livers after portal vein perfusion with collagenase collagenase type II (Worthington) in GBSS (PAN Biotech), followed by in vitro digestion with collagenase (type II, Worthington) in a rotatory water bath at 37 °C and density gradient centrifugation. Liver sinusoidal endothelial cells (LSECs) were then obtained by

immunomagnetic separation using anti-CD146 coated microbeads (Miltenyi biotec) reaching a purity of ≥95%. LSECs were cultured in collagen coated flat-bottom 96 well microplates until they reached confluence for 48 h after isolation, and after careful medium exchange LSECs were then used for experiments.

Ex vivo stimulation/treatment
T cells were cultivated in RPMI-1640 medium (GIBCO) supplemented with 10% FCS, 1% L-Glutamine (200 mM), 1% Penicillin/Streptomycin (5000 U/mL), 50 µM 2-mercaptoethanol. For ex vivo stimulation and intracellular cytokine staining, cells were stimulated with 10 nM recombinant OVA peptide (SIINFEKL, peptides&elephants GmbH), HBV core peptide (MGLKFRQL, peptides&elephants GmbH) or 1x eBioscienceTM Cell stimulation cocktail (Thermo Fisher Scientific) together with 3 µg/mL Brefeldin A (Invitrogen). To analyse cAMP signalling, T cells were incubated for 1 h with the adenylyl cyclase agonist Forskolin (25 µM, Sigma-Aldrich), the PKA agonist Sp-8br-cAMPS (250 µM, Cayman Chemical), the EPAC agonist 8-pCPT-2'-O-Me-cAMP (30µM, Tocris), or the adenosine A2A receptor agonist CGS21680 (100 nM, Tocris) solved in DMSO (Sigma-Aldrich).

PBMC isolation from patients:
Venous blood samples were collected in EDTA-coated tubes. PBMCs were isolated by density gradient centrifugation using lymphocyte separation medium (PAN Biotech). Isolated PBMCs were resuspended in RPMI 1640 medium supplemented with 10% FCS, 1% penicillin/streptomycin and 1.5% 1 M HEPES buffer (ThermoFisher) and stored at -80 °C until used. Frozen PBMCs were thawed in complete medium (RPMI 1640 supplemented with 10% FCS, 1% penicillin/streptomycin and 1.5% 1 M HEPES buffer (ThermoFisher)) containing 50 U ml−1 benzonase (Sigma).

Surface stainings were performed at 4° C for 30 min. MHC class I H-2KbSIINFEKL-restricted or H-2KbMGLKFRQL -restricted streptamers for staining of murine T cells (Nauerth et al., 2016, DOI: 10.1002/cyto.a.22933) were kindly provided by D. Busch (Institute of Microbiology, TUM). For labelling, 0.4 µg streptamer per sample were incubated with 0.4 µL Strep-Tactin-PE/APC (IBA lifesciences) in PBS for 30 min on ice prior to incubation with cell suspensions.
HLA class I epitope-specific tetramers for staining of human T cells were generated through conjugation of biotinylated peptide/HLA class I monomers with PE-conjugated streptavidin (ProZyme, USA) at a peptide/HLA I:streptavidin molar ratio of 5:1.
To exclude dead cells, Fixable Viability Dye eFluor780 (Invitrogen) was included in the staining panels. For intracellular staining of cytokines, IC fixation buffer (Invitrogen) was used according to the manufacturer´s instructions. Staining of Granyzme B and Tox was performed in combination with Foxp3 / Transcription Factor staining Buffer set (Thermo Fisher Scientific) according to the manufacturer´s instructions. For staining of Crem and pPKA, cells were fixed in IC fixation buffer (Invitrogen) for 30 min and permeabilized with ice-cold Methanol for 30 min before staining.

Sample preparation for bulk RNA sequencing of OVA257-264-specific CD45.1+ CD8 T cells: Liver-associated lymphocytes and splenocytes from mice with resolved Ad-CMV-GOL infection were sorted into CD45.1+CXCR6+CX3CR1negCD8 and CD45.1+CXCR6negCX3CR1+CD8 T cells. CD8 T cells derived from mice with persistent Ad-TTR-GOL infection were sorted into CXCR6+CX3CR1negCD45.1+CD8 and CXCR6+CX3CR1+CD45.1+CD8 populations. 5000 cells per sample were collected in 1 x TCL lysis buffer (Qiagen) supplemented with 1% (v/v) 2-mercaptoethanol and immediately frozen on dry ice.

Sample preparation for bulk RNA sequencing of P14 LCMV-specific CD8 T cells: P14 cells were adoptively transferred into C57BL/6 mice and infected one day later with either LCMV Clone 13 or LCMV Armstrong. Resident (CD69+CD101+CXCR6+CX3CR1neg) and effector/effector-memory (CX3CR1+) P14 cells from the liver were sorted at d27 p.i. . Total RNA was isolated using the RNAdvance Cell v2 kit (Beckmann-Coulter).

Sample preparation for bulk RNA sequencing of Cor93-100-specific CD45.1+CD8 T cells: Liver-associated lymphocytes and splenocytes from mice with Ad-HBV infection were pre-gated on (CD19/Ly6G/TER119/CD335)neg CD8 T cells and sorted into liver CXCR6+CD45.1+, liver CD45.1+CX3CR1+ CD8 T cells, spleen CD45.1+CX3CR1+ CD8 T cells and liver CD45.1neg CD8 T cells from resolved infections and liver CD45.1+CXCR6+ and liver CD45.1+CXCR6+CX3CR1+ CD8 T cells and liver CD45.1neg CD8 T cells from persistent infection. 100 CD8 T cells were directly sorted into 96 well plates prepared with 1X Reaction Buffer consisting of lysis buffer and RNase Inhibitor for low input RNA sequencing (Takara). Plates were spun down and immediately stored on dry ice or at -80°C until further processing.

Sample preparation for scRNAseq of human HBcore-specific CD8 T cells: HBVcore18-specific CD8 T cells were enriched by magnetic bead-based sorting, and surface staining was performed. In total, 1152 live HBVcore18-specific CD8 T cells were sorted in 384-well plates (Bio-Rad) containing lysis buffer and mineral oil using FACS Melody Cell Sorter in single-cell sorting mode. Naive CD45RA+CCR7+ T cells were excluded.

scRNAseq of human HBV-specific CD8 T cells isolated from the liver by fine needle aspiration: Cells were thawed and stained with lineage marker antibodies as well as HBV multimers for two distinct HBV-specificities. The live HBV-specific CD8 T cells were sorted in 96-well Armadillo plates (Thermo Fisher Scientific) containing RNA lysis buffer using a BD SORP FACS Aria in index single-cell sorting mode.

| | |
|---|---|
| Instrument | Sony SP6800 spectral analyzer (Sony Biotechnology) and CytoFLEX S (Beckman Coulter) |
| Software | Data collection was performed with the SP6800 software (Sony Biotechnology) and analysed with FlowJo v10.7.1 & v10.8.0 (BD), R v4.0.2 and R cytofkit GUI v0.99 |
| Cell population abundance | Samples were sorted using the purity mode. Purity check post sorting confirmed >95% purity. |
| Gating strategy | FSC-A/SSC-A (Lymphocytes) -> FSC-W/FSC-H (Singlets) -> live-dead/autofluorescence (viable autofluorescence-negative) -> T cell stainings |

☒ Tick this box to confirm that a figure exemplifying the gating strategy is provided in the Supplementary Information.
